# Assessing two methods for estimating excess mortality of chronic diseases from aggregated data

**DOI:** 10.1186/s13104-020-05046-w

**Published:** 2020-04-10

**Authors:** Ralph Brinks, Thaddäus Tönnies, Annika Hoyer

**Affiliations:** 1 Institute for Biometry and Epidemiology, German Diabetes Center, Auf’m Hennekamp 65, 40225 Duesseldorf, Germany; 2 Department and Hiller Research Unit for Rheumatology, University Hospital Duesseldorf, Moorenstr. 5, 40225 Duesseldorf, Germany

**Keywords:** Chronic disease epidemiology, Multi-state model, Prevalence, Incidence, Dementia, Diabetes, Partial differential equation

## Abstract

**Objective:**

To assess the numerical properties of two recently published estimation techniques for excess mortality based on aggregated data about diabetes in Germany.

**Results:**

Application of the new methods to the claims data yields implausible findings for the excess mortality of type 2 diabetes in ages below 50 years of age.

## Introduction

Aggregated data such as health insurance claims data become more and more available for research purposes. Recently, we proposed a new method to estimate the excess mortality in chronic diseases from aggregated age-specific prevalence and incidence data [1, 2]. So far, estimates of excess mortality have only been plausible for ages 50+ and have shown to be unstable in younger ages. For example, in the simulation study of [2], the bias increases as the age decreases (Table 1 in [2]).

The theoretical background for estimating the excess mortality stems from the illness-death model for chronic diseases [3]. In [4] we have shown that the temporal change, ∂*p* = (∂_t_ + ∂_a_) *p* of the age-specific prevalence *p* is related to the incidence rate *i*, the mortality rates *m*_0_ and *m*_1_ of the people with and without the disease, respectively, the general mortality *m* and the mortality rate ratio *R* = *m*_1_/*m*_0_ via the following equations:

1a $$ {\partial p} = \, \left( {{ 1 { }{-}p}} \right) \, \{{ i{-}}p \times \left( {{m_{ 1} {-}m_{0}} } \right)\} $$

1b $$ = \left( { 1 { }{-}p} \right)\{ i{-}m \times p\left( {R{-}{ 1}} \right)/\left[ { 1 { } + p\left( {R{-}{ 1}} \right)} \right]\} . $$

There are two assumptions such that Eqs. (1a) and (1b) are true: (a) there is no remission from the chronic condition back to the healthy state and (b) age-specific prevalence of the chronic condition in the migrating population is the same as in the resident population.

Given the age-specific prevalence *p*, the age-specific incidence rate *i* and the general mortality rate *m*, Eqs. (1a) and (1b) can be used to estimate the excess mortality rate ∆*m* = *m*_1_ − *m*_0_ and the mortality rate ratio *R* [1, 2]:

2a $$ \Delta m = \, \{ i{-}\partial p/\left( { 1 { }{-}p} \right)\} /p, $$

2b $$ R = { 1 } + { 1}/p \times \{ i\left( { 1 { }{-}p} \right) \, {-}\partial p\} /\{ \left( { 1 { }{-}p} \right) \, \left( {m{-}i} \right) \, + \partial p\} $$

The aim of this research note is to explore the reasons why estimates of excess mortality for younger ages are biased and what can be done to extend the age range to ages below 50 years. As a testing example, we use claims data about diabetes from the German statutory health insurance based on about 70 million people collected during the period from 2009 to 2015 [5].

## Main text

### Methods and materials

Goffrier et al. report the age-specific prevalence *p* of type 2 diabetes in 2009 and 2015 [5]. The age-specific prevalence data *p* for men in 2009 and 2015 are modeled by a linear regression model after application of a logit transformation. Furthermore, the age-specific incidence rate *i* for diabetes in men halfway between 2009 and 2015, i.e., in the year 2012, is reported. The age-specific incidence rate *i* for 2012 is modeled by a linear regression model after a log-transformation. These data are used as input for Eqs. (2a) and (2b). For applying Eq. (2b) we also use the general mortality *m* in 2012 from the Federal Statistical Office of Germany.

With these input data, Eqs. (2a) and (2b) allow to estimate the age-specific excess mortality ∆*m* and the mortality rate ratio *R*. While *R* has a straightforward interpretation as the ratio of the mortality rate of the diabetic population compared to the non-diabetic population, the excess mortality rate ∆*m* is more interpretable when it is related to another mortality rate. As it holds *m* = *p m*_1_ + (1 − *p*) *m*_0_, we have ∆*m*/*m *≤ ∆*m*/*m*_0_=* R* − 1 and thus *R* ≥ 1 + ∆*m*/*m* ≥ ∆*m*/*m*. Hence, we decided to report the quotient ∆*m*/*m*, which is a lower bound for *R*.

In order to assess uncertainty in the results, we implemented a multidimensional probabilistic sensitivity analysis [6]. The key idea is to randomly sample from the distributions of input parameters (i.e., prevalence in 2009 and 2015, and incidence in 2012), and calculate the outcomes (i.e., measures of excess mortality). As the input parameters are sampled from random distributions many times, we get a sequence of outcomes, which also follows a random distribution representing the combined uncertainty in the input parameters [6]. We report empirical medians, and 2.5% and 97.5% quantiles for approximate 95% confidence intervals of the outcomes based on 5000 samples from the input distributions.

### Results

**Fig. 1:**
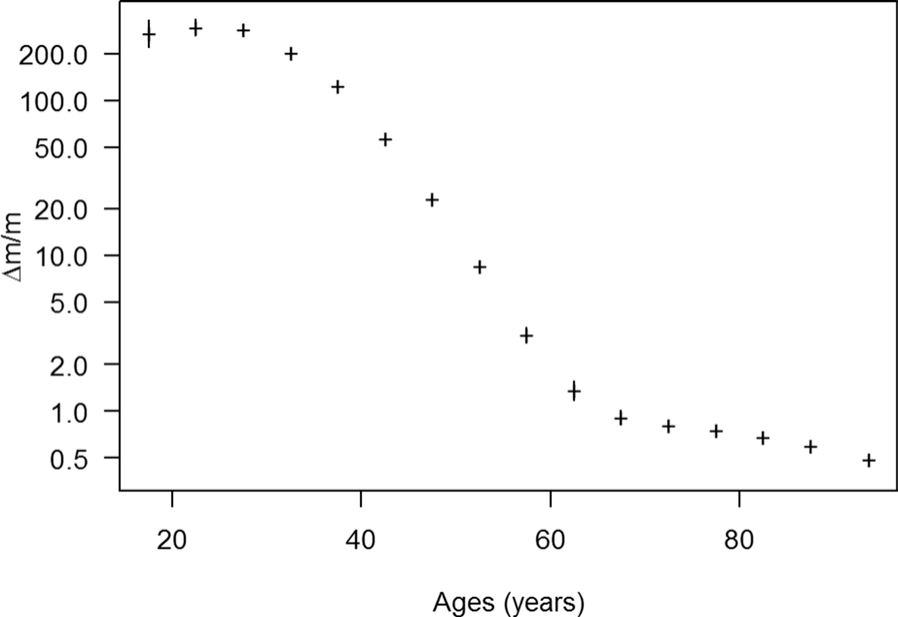
Age-specific ratio ∆*m*/*m* of the excess mortality (∆*m*) and the general mortality (*m*). The graph shows the empirical median of ∆*m*/*m* with 95% confidence bounds (vertical bars) based on the probabilistic sensitivity analysis with 5000 simulation runs. The ratio ∆*m*/*m* is a lower bound for the mortality rate ratio *R*

Figure 1 shows the age-specific ratio ∆*m*/*m*. Below 50 years of age the excess mortality rate is more than 10 times higher than the mortality rate of the general population. The ratio peaks at a value of more than 200 at the age of about 30 years. As *R* ≥ ∆*m*/*m*, we see that the estimate of the excess mortality is extraordinarily high.

Application of Eq. (2b) for obtaining the mortality rate ratio *R*, yields the results as shown in Table 1. We see that for ages below 55 years of age, the mortality rate ratios are implausibly high or become negative. By definition of the mortality rate ratio, a quotient of two positive rates, negative values are not possible. Thus, we see that the estimates based on Eq. (2b) do not yield sensible results for lower age groups and thus are not reliable.

**Table 1:** Mortality rate ratios (*R*) for different age-groups

Age group (years)	Mortality rate ratio *R* (median, 95% confidence bounds)
15–19	395 [294, 522]
20–24	810 [592, 1159]
25–29	− 1610 [− 3055, − 1132]
30–34	− 335 [− 368, − 309]
35–39	− 140 [− 145, − 135]
40–44	− 90.9 [− 93.8, − 86.6]
45–49	− 119 [− 150, − 100]
50–54	35.5 [27.8, 47.8]
55–59	6.45 [5.48, 7.65]
60–64	2.87 [2.53, 3.25]
65–69	2.19 [2.03, 2.37]
70–74	2.08 [1.99, 2.18]
75–79	2.02 [1.97, 2.06]
80–84	1.89 [1.86, 1.91]
85–89	1.73 [1.71, 1.74]
90+	1.55 [1.53, 1.57]

### Discussion

In this manuscript we have applied two methods to estimate indices for the excess mortality of a chronic condition from age-specific prevalence and incidence data. The first index is the difference ∆*m* between the mortality rate of the diseased people (*m*_1_) and the people without the disease (*m*_0_), i.e., ∆*m *= *m*_1_ − *m*_0_. Sometimes, the index ∆*m* is called attributable risk [7]. The second index is the mortality rate ratio *R* = *m*_1_/*m*_0_. In an example about diabetes in the German male population, it turns out that both estimates are numerically unstable for ages below 50 years. In case of ∆*m*, unreasonably high values have been obtained in the diabetes data (more than 200 times the mortality of the general population). The estimated values of *R* can lead to implausible results such as negative rate ratios.

The question arises if the implausible results might be a consequence of the assumptions for Eq. (1) being violated. The two assumptions are: no remission and prevalence in migrants is the same as in residents. While remission of diabetes has indeed been observed [8], it has not been a relevant therapy option or health policy in Germany during the study period. Note that the input data [5] refers to millions of people. Little is known about the second assumption. The prevalence of diabetes in migrants from and to Germany is currently not investigated on population level. However, in another age-related chronic disease (dementia), we analyzed the most extreme cases (i.e., all immigrants having the chronic condition and all emigrants being free from the chronic condition and vice versa) and the overall epidemiological measures were only negligibly affected [9]. Thus, we think that violations of the two assumptions have only very minor effects on reported results.

Implausible results, at least in theory, may be due to changes in the distributions of relevant covariates in the input data. Examples for relevant covariates might be the change of diagnostic criteria for diabetes, changes in the distribution of disease duration, distribution of body weight, the quality of glucose control or the presence of co-morbidities. In fact, possible effects of changing covariates are not estimable by our method and we do not doubt that these exist. However, we believe that the study period (2009–2015) is relative short to comprise considerable changes. Furthermore, in Germany there has not been a change in diagnostic criteria in diabetes during the study period.

In simulation studies, we found that the diagnostic accuracy of the claims data plays a crucial role for the proposed methods of estimating excess mortality. By diagnostic accuracy we mean sensitivity and specificity of the claims data compared to the gold standard of diagnosing diabetes. In principle, diagnostic accuracy may undergo secular changes, e.g., if reimbursement policy is changing. It could be possible, for instance, that false positive diagnoses in the prevalence of 2015 can be increased compared to 2009, if physicians obtain more reimbursement for the later point in time. We note, however, that such up-coding is fraud and is enforced by penalty. The impact of changes of diagnostic accuracy is subject to an ongoing theoretical analysis (including a comprehensive simulation study) aimed for an upcoming paper.

Based on the results in this example, we see that special attention is required in interpreting the results of the two estimation techniques, when applied to lower age ranges.

## Limitations

The aim of this research note was to assess the performance of two recently published estimators for the excess mortality of a chronic disease from prevalence and incidence data. While in previous publications [1, 2] reasonable results have been found for ages over 50 years, here we demonstrated problems of these estimators in younger age groups. The reasons for the problems seem to lie in the estimators itself. For instance, if the partial derivative of the prevalence (∂*p*) is close to zero and the incidence rate (*i*) is close to the general mortality (*m*), i.e., *i* ≈ *m*, the denominator in Eq. (2b) is close to zero. Thus, the fraction on the right hand side of Eq. (2b) becomes very large in magnitude. This explains the highly oscillating values in Table 1. Despite Eq. (2a) does not have the (cancellation) problem for *i* ≈ *m*, implausibly high values are obtained too. The reason is the factor 1/*p* on the right hand side of Eq. (2a). For values of the prevalence (*p*) being close to zero, the reciprocal 1/*p* becomes very large. For example, in the lowest age group (15–19 years), the fraction 1/*p* takes values of about 900, which explains the high estimate for ∆*m* in this age group. Strategies to overcome these problems are currently under development and will be subject of a future article.

## Supplementary information

**Additional file 1.** Script (plain text file, accessible via any text editor, e.g., Notepad, GNU Emacs etc.) for the example about type 2 diabetes, intended to use with the statistical software R (The R Foundation of Statistical Software).

## Data Availability

The source code for this analysis is available as an electronic supplement to this published article (Additional file 1). The underlying data about diabetes were taken from a free publicly available source [5], which has been cited in the text and is part of the source code (see Additional file 1).
